# Clinical Correlates of Health-Related Quality of Life in Adults With Chronic Tic Disorder

**DOI:** 10.3389/fpsyt.2021.619854

**Published:** 2021-03-10

**Authors:** David A. Isaacs, Heather R. Riordan, Daniel O. Claassen

**Affiliations:** ^1^Department of Neurology, Vanderbilt University Medical Center, Nashville, TN, United States; ^2^Department of Pediatrics, Monroe Carell Jr. Children's Hospital at Vanderbilt, Nashville, TN, United States

**Keywords:** Tourette syndrome, tic disorder, psychiatric comorbidities, premonitory urge, quality of life, health related quality of life

## Abstract

Tics are the hallmark feature of Tourette syndrome (TS), but psychiatric and sensory symptoms are widely prevalent and increasingly recognized as core manifestations of the disorder. Accumulating evidence suggests that these psychiatric and sensory symptoms exert greater influence on quality of life (QOL) than tics themselves. However, much remains uncertain about determinants of QOL in TS due to the complexity of the clinical presentation. Here, we sought to clarify the association between health-related QOL (HRQOL) and common psychiatric and sensory symptoms in adults with TS and other chronic tic disorders. To do so, we prospectively recruited 52 patients from a tertiary care clinic to complete self-report measures assessing HRQOL (Gilles de la Tourette-Quality of Life Scale, GTS-QOL), depression (Patient Health Questionnaire-9, PHQ-9), anxiety (Generalized Anxiety Disorder Scale-7, GAD-7), obsessive-compulsive symptoms (Dimensional Obsessive-Compulsive Scale, DOCS), attention deficit hyperactivity disorder symptoms (Adult ADHD Self-Report Screening Scale for DSM-5, ASRS-V), and premonitory urge (Premonitory Urge to Tic Scale, PUTS). All participants were also administered the Yale Global Tic Severity Scale (YGTSS) to quantify tic severity. Using correlational analysis and multivariable linear regression modeling, we found that GTS-QOL score was significantly associated with scores from all other rating scales, with the exception of the PUTS. GTS-QOL was most strongly associated with PHQ-9, followed by ASRS-V, GAD-7, DOCS, and YGTSS total tic score. The regression model including these five independent variables, as well as sex, explained 79% of GTS-QOL score variance [*F*_(6,40)_ = 29.6, *p* < 0.001]. Specific psychiatric symptoms differentially impacted physical, psychological, and cognitive HRQOL. Systematic assessment of psychiatric comorbidities is imperative for clinical care and clinical research efforts directed at improving QOL in adults with chronic tic disorders.

## Introduction

Tourette syndrome (TS) is a multi-faceted neurodevelopmental disorder affecting 0.3–1% of school-aged children (1, 2), and one-third of these patients continue to experience bothersome tics into adulthood (3). Tics are the defining characteristic of TS, but psychiatric and sensory symptoms are increasingly recognized as core manifestations. Ninety-percent of TS patients have at least one comorbid psychiatric diagnosis, and 60% have two or more (4). Obsessive-compulsive disorder (OCD) and attention deficit hyperactivity disorder (ADHD) are the two most common psychiatric comorbidities in TS, with respective prevalence ranges of 10–50% and 30–60% (4–7). Features of OCD and/or ADHD are also frequently evident among TS patients not fulfilling diagnostic criteria for these disorders (8). Depression and anxiety are common, occurring in 30% and 36% of TS patients, respectively (4). The prevalence of several other psychiatric conditions, including impulse control disorder and autism spectrum disorder, is elevated in TS samples relative to the general population (4, 6, 9, 10). In addition to psychiatric comorbidities, 90% of adult and adolescent patients endorse pre-monitory urges, which are uncomfortable sensory disturbances preceding tics (11, 12).

Psychiatric and sensory symptoms of TS undermine patient quality of life (QOL) (12–16). In children, ADHD (15, 17–19), OCD (15, 17, 18), and depression (15) are each linked with worse QOL. In adults, depression appears to be the predominant determinant of poor QOL (20–23), though OCD (20), anxiety (24), and premonitory urge (11) are also implicated. QOL is lowest in those patients afflicted with multiple psychiatric comorbidities (19). Mounting data suggests that psychiatric (15, 19, 20, 24, 25) and sensory (26) symptoms exert greater influence on QOL than tics themselves. Studies exploring QOL in TS vary considerably in their clinical assessments; QOL measures [generic (15, 17, 18, 20–23, 25, 27) vs. disorder-specific QOL (11, 24)]; sample populations [treatment-seeking (11, 15, 17, 18, 20, 21, 23, 24) vs. non-treatment-seeking (22, 25, 27), children (15, 17, 18, 24) vs. adults (11, 20–23, 25, 27)]; and statistical methodologies. QOL research in adults with TS has focused on the roles of depression (11, 19–21, 23, 24), anxiety (11, 19, 20, 23, 24), and OCD (11, 19, 20, 23), with lesser attention devoted to ADHD (11, 19), other psychiatric diagnoses (19), and premonitory urge (11, 24). Given their frequent co-occurrence and co-variance, neglecting one or more of these symptoms in an analysis could inflate or deflate the statistical relevance of factors under consideration, leading to potentially inaccurate conclusions (28). Thus, despite the expanding literature on QOL in TS, important ambiguities remain, in large part due to the complexity of the TS phenotype and the multidimensionality of the QOL construct. When gauging the QOL impact of health or disease, researchers typically consider health-related QOL (HRQOL), conceptualized as the perceived influence of physical, emotional, and social health on an individual's overall satisfaction with life (29). Enhanced understanding of HRQOL determinants in TS is vital in order to direct attention and treatment to those factors most distressing to patients.

In this study, we sought to clarify the association between HRQOL and common psychiatric and sensory symptoms in adults with TS and other chronic tic disorders. To do so, we prospectively recruited patients from a specialty TS clinic to undergo clinical rating of tic severity and to complete a battery of self-report measures assessing depression, anxiety, obsessive-compulsive symptoms, ADHD symptoms, premonitory urge, and HRQOL.

## Materials and Methods

### Participants

From April 2019 through September 2020 we consecutively recruited adults with TS or other chronic tic disorders from Vanderbilt University Medical Center (VUMC) Tourette Syndrome Clinic and from institutional research registries. Inclusion criteria included diagnosis of TS, chronic motor tic disorder, or chronic vocal tic disorder based on Diagnostic and Statistical Manual of Mental Disorders, 5th edition (DSM-5) criteria, age ≥ 18 years, capacity to provide informed consent, and ability to speak English. For the purposes of this article, the term “chronic tic disorder” is used to encompass TS, chronic motor tic disorder, and chronic vocal tic disorder. Participants gave electronic informed consent prior to performing study-specific procedures. The study was conducted in accordance with the Declaration of Helsinki and was approved by the VUMC Institutional Review Board.

### Measures

An experienced movement disorders neurologist (D.I.) administered the YGTSS to all participants. Subsequently, participants completed a battery of validated self-report instruments assessing common psychiatric and sensory symptoms in TS, including depression, anxiety, obsessive-compulsive symptoms, ADHD symptoms, and premonitory urge. Table 1 summarizes properties of these scales. In addition, participants completed the Gilles de la Tourette-Quality of Life Scale (GTS-QOL), a disorder-specific scale developed to assess QOL domains most impacted by TS (39). The GTS-QOL contains 27 items, each with a five-point Likert scale ranging from 0 (“no problem”) to 4 (“extreme problem”). As previously identified by exploratory factor analysis, the scale is composed of four sub-scales: Psychological (11 items), Physical/Activities of Daily Living (Physical/ADL) (7 items), Obsessive-Compulsive (OC) (5 items), and Cognitive (4 items) (39). Item scores within each subscale are summed and then normalized to 100 to generate the subscale score. The four subscale scores are then summed and normalized to 100 to yield the total score. The GTS-QOL has undergone rigorous development and validation, demonstrating satisfactory internal consistency, test-retest reliability, construct validity, and convergent and discriminant validity (39).

**Table 1:** Clinical rating scales.

**Scale name**	**#of scale items**	**Scale range**	**Score interpretation**	**Additional notes on scale properties**
YGTSS Total Tic Score (TTS) (30)	10	0–50	Higher score indicates more severe tics	Gold-standard, semi-structured clinician-administered interview for rating tic severity. The scale is composed of two subscales: motor tic scale (0–25) and phonic tic scale (0–25).
Premonitory Urge to Tic Scale (PUTS) (31)	10	9–36	Higher score indicates more severe premonitory urge	Scale item 10 concerns tic suppressibility and is not included in total score as it measures a separate construct from the other scale items (31).
Dimensional Obsessive-Compulsive Scale (DOCS) (32)	20	0–80	Higher score indicates more obsessive-compulsive symptoms	In clinical populations, DOCS total score cutoff ≥ 21 is 70% sensitive and 70% specific in distinguishing OCD from other anxiety disorders (32).
Adult ADHD Self-Report Screening Scale for DSM-5 (ASRS-V) (33)	6	0–24	Higher score indicates more ADHD symptoms	In clinical populations, ASRS-V total score cutoff ≥ 14 is 81% sensitive and 70% specific for detecting ADHD (34).
Generalized Anxiety Disorder-7 (GAD-7) (35)	7	0–21	Higher score indicates more anxiety	GAD-7 shows good reliability, convergent validity, and discriminant validity in individuals with anxiety and mood disorders (36).
Patient Health Questionnaire-9 (PHQ-9) (37)	9	0–27	Higher score indicates more depression	PHQ-9 shows good reliability, convergent validity, and discriminant validity in psychiatric settings (38).
Gilles de la Tourette Syndrome-Quality of Life Scale (GTS-QOL) (39)	27	0–100	Higher score indicates worse HRQOL	The scale comprises 4 sub-scales: psychological, physical/ADL, obsessive-compulsive, and cognitive. Raw scores are normalized to 100.

Self-report measures were administered in Research Electronic Data Capture (REDCap), a secure, web-based platform for collecting patient-reported information and storing research data (40, 41). Participants were permitted to complete online self-report scales in clinic or at home, as per their preference. Median time between administration of the YGTSS and completion of the self-report scale battery was 6.5 days (interquartile range 0–13.25 days).

### Statistical Analysis

Measures of central tendency were calculated for relevant clinical variables. Spearman's rank correlation was used to assess degree of relationship between clinical measures. Significance thresholds were Bonferroni-adjusted to correct for multiple comparisons in the correlational analysis, resulting in a pre-specified acceptable Type 1 error rate of 0.001 (45 total comparisons in correlational analysis).

To determine the independent association of specific clinical variables with GTS-QOL total score, we conducted a hierarchical linear regression analysis with backwards elimination. Clinical variables that did not significantly correlate with GTS-QOL total score were not included in this regression analysis. As such, sex, YGTSS total tic score (TTS), DOCS score, ASRS-V score, PHQ-9 score, and GAD-7 score were included as independent variables in the first regression model of the analysis. The sample size was inadequate to permit inclusion of interaction terms. To identify influential outliers, we calculated Cook's D values for all observations in the first model; observations with Cook's *D-*value > 4/n were excluded from the regression analysis. Regression diagnostics performed at each step of the analysis included assessment for multicollinearity (conservatively defined as variance inflation factor > 2.5), normality of residuals (with Shapiro-Wilk-test of model residuals), heteroskedasticity (with Breusch-Pagan-test), model explanatory power (indexed by adjusted *R*^2^), and model quality [by Akaike information criteria, with lower values indicating superior model quality (42)]. At each step in the hierarchical analysis, we identified and removed the independent variable with the largest standardized coefficient. F-statistics were calculated to compare change in non-adjusted *R*^2^ following stepwise removal of independent variables. The acceptable Type 1 error rate for explanatory power of the models was set at 0.01 to account for multiple comparisons (5 models were constructed with GTS-QOL total score as the dependent variable). *Post-hoc t*-tests were performed to compare each independent variable in the model to GTS-QOL total; significance thresholds for *post-hoc* tests were Bonferroni-adjusted to correct for multiple comparisons. Both non-standardized and standardized independent variable coefficients (β) are reported.

To determine the independent association of clinical variables with GTS-QOL sub-scales, we performed separate multivariable linear regression analyses. Only those variables significantly associated with GTS-QOL total score, as per the hierarchical regression analysis, were included. The aforementioned regression diagnostics were undertaken for each model. The acceptable Type 1 error rate for model explanatory power was set at 0.025 to account for multiple comparisons (2 models were constructed for each GTS-QOL subscale).

Data was complete for all measures except GAD-7. One patient omitted answers to two items for that scale. Missing values were imputed with predictive mean matching. Statistical analyses were conducted in STATA.

## Results

**Figure 1:**
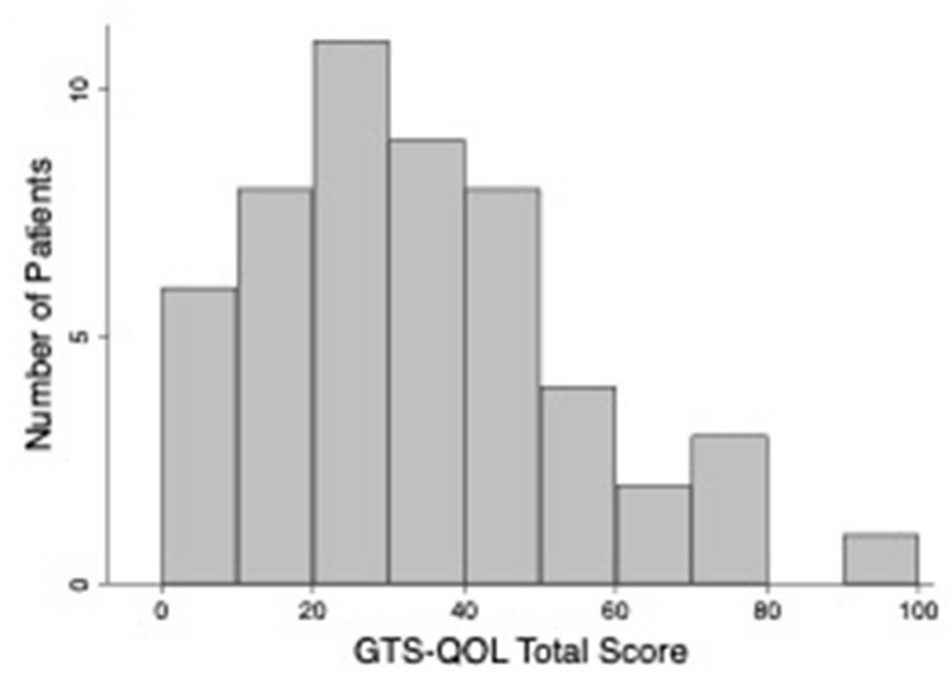
Histogram of GTS-QOL total scores.

Table 2 presents clinical characteristics of the study population. The sample included three participants with chronic motor tic disorder; all other participants met criteria for TS. Eighty-nine percent of participants reported an established diagnosis of at least one of the following: depression, anxiety, OCD, ADHD, autism spectrum disorder, or impulse control disorder; 71% of participants reported 2 or more of these diagnoses. Forty-eight percent screened positive for ADHD, and the same percentage screened positive for OCD, based on validated scale cutoffs (see Table 1). Table 3 displays measures of central tendency for each rating scale. Figure 1 depicts the distribution of GTS-QOL total scores within the study population.

**Table 2:** Cohort clinical characteristics.

Sex (M:F)	35:17
Age (years)	33 (22–47.5)^∧^
**Ethnicity**	
Hispanic or Latino	0
Not Hispanic or Latino	52
Unknown/not reported	0
**Race**	
Asian	1
Black or African American	0
White	50
More than one race	1
**Chronic tic disorder diagnosis**	
Tourette syndrome	49
Chronic motor tic disorder	3
Chronic vocal tic disorder	0
**Self-reported history of:**	
OCD	25
ADHD	12
Anxiety	35
Depression	31
Autism spectrum disorder	1
Impulse control disorder	6
None of above	6
**Self-reported current use of psychotropic medications:**	
None	13
SSRI and/or SNRI	24
Benzodiazepine	14
Antipsychotic	14
Mood stabilizer^+^	8
α-agonist	6
Stimulant	6
Other psychotropic medications	22

∧ *Median (interquartile range, IQR)*.

+ *Mood stabilizers in this sample included lithium, lamotrigine, and/or oxcarbazepine*.

*SSRI, selective serotonin reuptake inhibitor; SNRI, serotonin-norepinephrine reuptake inhibitor*.

**Table 3:** Clinical rating scale scores.

**Scale**	**Score^∧^**
TTS	25.5 (15–32)
PUTS	25 (21.5–28)
DOCS	20 (12–33)
ASRS-V	13 (11–16)
GAD-7	10 (5.5–15)
PHQ-9	10.5 (5–15)
GTS-QOL Total	30.4 (19.5–47.2)
GTS-QOL Psychological sub-scale	35.2 (19.3–56.8)
GTS-QOL Physical/ADL sub-scale	26.8 (10.7–46.4)
GTS-QOL OC sub-scale	20.0 (10.0–35.0)
GTS-QOL Cognitive sub-scale	37.5 (18.8–56.3)

∧ *Median (IQR)*.

The correlation matrix for the study rating scales is presented in Table 4. After correcting for multiple comparisons, GTS-QOL total positively correlated with scores from all other scales except PUTS. GTS-QOL total correlated most strongly with PHQ-9 and GAD-7, followed by ASRS-V, DOCS, and TTS. GTS-QOL Psychological, Physical/ADL, and Obsessive-Compulsive sub-scale scores likewise positively correlated with scores from all measures except PUTS. GTS-QOL Cognitive sub-scale score correlated only with PHQ-9 and ASRS-V scores.

**Table 4:** Spearman rank correlation matrix.

	**TTS**	**PUTS**	**DOCS**	**ASRS-V**	**PHQ-9**	**GAD-7**
PUTS	0.34					
DOCS	0.40	0.36				
ASRS-V	0.38	0.19	0.34			
PHQ-9	0.25	0.25	0.50^**^	0.54^**^		
GAD-7	0.50^*^	0.29	0.55^**^	0.31	0.53^*^	
GTS-QOL Total	0.55^**^	0.36	0.60^**^	0.61^**^	0.71^**^	0.66^**^
GTS-QOL-Psych	0.45^*^	0.32	0.51^*^	0.50^*^	0.70^**^	0.70^**^
GTS-QOL-Phys/ADL	0.68^**^	0.41	0.60^**^	0.51^*^	0.59^**^	0.63^**^
GTS-QOL-OCD	0.59^**^	0.44	0.66^**^	0.46^*^	0.61^**^	0.61^**^
GTS-QOL-Cognitive	0.31	0.18	0.39	0.70^**^	0.59^**^	0.38

* p < 0.001;

** *p < 0.0001*.

Table 5 provides details of the models from the hierarchical regression analysis for GTS-QOL total. Following construction of the first model, observations from four participants were identified as outliers and excluded from the analysis. PHQ-9 and ASRS-V were independently associated with GTS-QOL total, even after controlling for sex and severity of tics, anxiety, and obsessive-compulsive symptoms (Model 1). With PHQ-9 and ASRS-V eliminated from the model, GAD-7 and DOCS were each independently associated with GTS-QOL (Model 3). Only after removal of PHQ-9, ASRS-V, GAD-7, and DOCS from the model was TTS significantly associated with GTS-QOL total (Model 5). PHQ-9, ASRS-V, GAD-7, DOCS, and TTS all contributed to model explanatory power, as evidenced by significant decrease in unadjusted *R*^2^ following stepwise elimination of these variables. Notably, Model 2 exhibited heteroskedasticity, and residuals in Models 2 and 5 were not normally distributed, indicating linear regression assumptions were partially violated in these two nested models.

**Table 5:** Hierarchical regression analysis with GTS-QOL total score as dependent variable.

**Model**	**IVs^+^**	**VIF for model IVs**	**Shapiro-Wilk test for normality of model residuals^&^**	**Breusch-Pagan test for hetero-skedasticity**	**Model *F*-value, Adj *R*^**2**^, AIC**	**Δ *R*^**2**^ compared to prior model**	**IVs that predict mean SGI score, non-standardized (standardized) coefficients^∧^**
1	Sex	1.15	*W* = 0.97 *p* = 0.20	χ^2^ = 2.23 *p* = 0.14	*F*_(6,41)_ = 29.6^**^ adj *R*^2^ = 0.79 AIC = 344	-	PHQ-9, β = 1.3 (0.45)^**^ ASRS-V, β = 1.5 (0.33)^*^
	TTS	1.54					
	DOCS	1.62					
	ASRS-V	1.44					
	PHQ-9	2.03					
	GAD-7	2.36					
2	Sex	1.08	*W* = 0.95 *p* = 0.04	χ^2^ = 5.03 *p* = 0.02	*F*_(5,42)_ = 20.9^**^ adj *R*^2^ = 0.68 AIC = 363	**Δ***R*^2^ = 0.10 *F*_(1,41)_ = 21.6^**^	ASRS-V, β = 2.1 (0.45)^**^ GAD-7, β = 1.2 (0.36)^*^
	TTS	1.43					
	DOCS	1.53					
	ASRS-V	1.28					
	GAD-7	1.79					
3	Sex	1.08	*W* = 0.98 *p* = 0.30	χ^2^ = 1.79 *p* = 0.18	*F*_(4,43)_ = 13.5^**^ adj *R*^2^ = 0.52 AIC = 382	**Δ***R*^2^ = 0.16 *F*_(1,42)_ = 23.0^**^	GAD-7, β = 1.2 (0.38)^*^ DOCS, β = 0.5 (0.37)^*^
	TTS	1.31					
	DOCS	1.45					
	GAD-7	1.79					
4	Sex	1.02	*W* = 0.97 *p* = 0.13	χ^2^ = 0.61 *p* = 0.44	*F*_(3,44)_ = 13.3^**^ adj *R*^2^ = 0.44 AIC = 388	**Δ***R*^2^ = 0.08 *F*_(1,43)_ = 7.9^*^	DOCS, β = 0.7 (0.53)^**^
	TTS	1.13					
	DOCS	1.11					
5	Sex	1.02	*W* = 0.92 *p* < 0.01	χ^2^ = 0.39 *p* = 0.53	*F*_(2,45)_ = 6.3* adj *R*^2^ = 0.18 AIC = 405	**Δ***R*^2^ = 0.26 *F*_(1,44)_ = 21.5^**^	TTS, β = 0.8 (0.43)^*^
	TTS	1.02					

+ *IV, independent variables*.

& *p-value > 0.05 signifies inability to reject null hypothesis that model residuals are normally distributed*.

∧ *Variables with lowest p-value listed first in each cell*.

* *p < 0.01*,

** *p < 0.001*.

In the separate regression analysis with respective GTS-QOL subscales as the dependent variable, sex was excluded as an independent variable since it was not significantly associated with GTS-QOL total at any step of the hierarchical regression analysis. GAD-7 exhibited mild multicollinearity (VIF between 2 and 2.5) in regression models with GTS-QOL subscales as the dependent variable. To address this, models with and without GAD-7 were constructed (see Table 6). Clinical measures were differentially associated with specific GTS-QOL subscales: GAD-7 and PHQ-9 with GTS-QOL Psychological; TTS with GTS-QOL Physical/ADL; and ASRS-V and PHQ-9 with GTS-QOL Cognitive. Residuals for the linear models of GTS-QOL Obsessive-Compulsive were not normally distributed, raising concern for model validity.

**Table 6:** Regression analyses with respective GTS-QOL subscales as dependent variable.

**GTS-QOL Subscale**	**Model**	**IV**	**#outliers removed**	**VIF for model IVs^&^**	**Shapiro-Wilk test for normality of model residuals^+^**	**Breusch-Pagan test for hetero-skedasticity**	**Model *F*-value** **Adj *R*^2^** **AIC**	**IVs that predict mean SGI score, non-standardized (standardized) coefficients^**∧**^**
Psychological	6	TTS	5	1.62	*W* = 0.99 *p* = 0.87	χ^2^ = 0.76 *p* = 0.38	*F*_(5,41)_ = 23.6^**^ adj *R*^2^ = 0.71 AIC = 375	GAD-7, β = 1.9 (0.45)^**^ PHQ-9, β = 1.34 (0.35)^*^
		DOCS		1.74				
		ASRS-V		1.59				
		PHQ-9		1.88				
		GAD-7		2.22				
	7	TTS	5^+^	1.35	*W* = 0.98 *p* = 0.66	χ^2^ = 0.43 *p* = 0.51	*F*_(4,42)_ = 19.5^**^ adj *R*^2^ = 0.65 AIC = 387	PHQ-9, β = 2.1 (0.53)^**^
		DOCS		1.59				
		ASRS-V		1.57				
		PHQ-9		1.51				
Physical/ADL	8	TTS	3	1.71	*W* = 0.95 *p* = 0.05	χ^2^ = 1.83 *p* = 0.18	*F*_(5,43)_ = 23.9^**^ adj *R*^2^ = 0.70 AIC = 384	TTS, β = 0.9 (0.43)^**^
		DOCS		2.08				
		ASRS-V		1.53				
		PHQ-9		2.08				
		GAD-7		2.29				
	9	TTS	3	1.36	*W* = 0.96 *p* = 0.12	χ^2^ = 1.45 *p* = 0.23	*F*_(4,44)_ = 28.5^**^ adj *R*^2^ = 0.70 AIC = 385	TTS, β = 1.1 (0.50)^**^ PHQ-9, β = 1.1 (0.30)^*^
		DOCS		1.90				
		ASRS-V		1.49				
		PHQ-9		1.73				
Obsessive-Compulsive	10	TTS	4	1.57	*W* = 0.82 *p* < 0.01	χ^2^ = 3.31 *p* = 0.069	*F*_(5,42)_ = 15.3^**^ adj *R*^2^ = 0.60 AIC = 358	DOCS, β = 0.42 (0.35)^*^
		DOCS		1.55				
		ASRS-V		1.42				
		PHQ-9		1.79				
		GAD-7		2.12				
	11	TTS	4	1.27	*W* = 0.82 *p* < 0.01	χ^2^ = 2.52 *p* = 0.11	*F*_(4,43)_ = 18.9^**^ adj *R*^2^ = 0.60 AIC = 357	DOCS, β = 0.47 (0.38)* TTS, β = 0.50 (0.32)^*^
		DOCS		1.41				
		ASRS-V		1.38				
		PHQ-9		1.42				
Cognitive	12	TTS	4	1.63	*W* = 0.99 *p* = 0.85	χ^2^ = 0.01 *p* = 0.90	*F*_(5,42)_ = 19.6^**^ adj *R*^2^ = 0.66 AIC = 389	ASRS-V, β = 2.99 (0.54)^**^ PHQ-9, β = 1.45 (0.38)^*^
		DOCS		1.83				
		ASRS-V		1.59				
		PHQ-9		1.80				
		GAD-7		2.26				
	13	TTS	4	1.41	*W* = 0.99 *p* = 0.86	χ^2^ = 0.00 *p* = 0.97	*F*_(4,43)_ = 24.6^**^ adj *R*^2^ = 0.67 AIC = 388	ASRS-V, β = 3.08 (0.55)^**^ PHQ-9, β = 1.28 (0.33)^*^
		DOCS		1.57				
		ASRS-V		1.54				
		PHQ-9		1.43				

& *Tested after removal of outliers*.

* *p < 0.01*,

** *p < 0.001*.

+ *Same outliers removed to permit model comparison*.

## Discussion

In this study, we examined the association of HRQOL with motor, psychiatric, and sensory symptoms in adults with chronic tic disorders. Our main finding is that psychiatric symptoms appear to be the predominant driver of HRQOL in this population. Novel findings include the significant association of ADHD with poor HRQOL and the differential impact of psychiatric symptoms on distinct HRQOL domains. We sequentially discuss these results and their ramifications for clinical care and research.

In this treatment-seeking sample of adults with chronic tic disorder, depression was most strongly linked with poor HRQOL, in accord with prior studies (20–22). The magnitude of depression's association with GTS-QOL total was twice as great as any other clinical factor. Anxiety, obsessive-compulsive symptoms, and ADHD symptoms were also independently associated with worse HRQOL. The relationship of the former two symptoms to HRQOL has been previously established (20, 23, 24); however, most investigations exploring QOL in adults with TS have not incorporated ADHD measures (20, 21, 23, 24). We were only able to find a single study with results suggesting that ADHD may negatively impact HRQOL in adults with chronic tic disorder (19). Methodologic limitations raise concerns regarding some of that study's conclusions: the sample population included both children and adults but the analysis was not stratified by age; and the analytic approach relied solely on reported diagnosis of ADHD (dichotomous variable) rather than severity of current ADHD symptoms (continuous variable) (19). Most investigations exploring QOL in adults with TS have not incorporated ADHD measures (20, 21, 23, 24). While inattention and hyperactivity symptoms tend to diminish with age in TS populations, half of patients meeting criteria for ADHD in childhood still meet criteria for the disorder as they enter adulthood (43). In a large international cohort study of 6,805 individuals with tic disorders, 61% of children and 39% of adults were diagnosed with ADHD (44). Outside the context of tic disorders, ADHD is increasingly recognized as a lifelong condition, with persistence of symptoms into adulthood for as many as two-thirds of individuals (45, 46). These adults with ADHD experience higher rates of workplace difficulties (47–49) and unemployment (47, 48), greater home life dysfunction (47, 48), and poor HRQOL (48). In children with tic disorders, the deleterious effect of ADHD on cognitive performance (50–53), psychosocial functioning (50, 54–56), and QOL (57, 58) has been repeatedly demonstrated, with several studies indicating ADHD exerts substantially greater impact on these domains than tics (50–55, 57). In adults with both ADHD and tic disorder, diagnosis of the former but not the latter accounts for impaired current and past global functioning (59). In our sample of adults with tic disorders, half of patients screened positive for ADHD, and ADHD symptoms were strongly associated with HRQOL, even after accounting for severity of depression, anxiety, and obsessive-compulsive symptoms. Patients with more severe ADHD symptoms endorsed poorer cognitive HRQOL, whereas no significant relationship emerged between ADHD symptoms and psychological, physical, or obsessive-compulsive HRQOL domains. Additional research is needed to further examine the course and role of ADHD symptomatology in adults with tic disorders, both in regards to QOL and functional impairment.

Study findings replicate prior work demonstrating QOL is more strongly associated with psychiatric symptoms than tics (20, 27). Greater tic severity did correspond with worse physical HRQOL, consistent with existing literature showing the detrimental physical toll of certain types of tics, particularly those causing pain (13, 16, 25). Severity of premonitory urge correlated with GTS-QOL total score in our study, but the significance of this relationship did not survive correction for multiple comparisons. Premonitory urge is a bothersome symptom for many patients (11), but our results indicate HRQOL is more tightly intertwined with psychiatric symptoms than pre-monitory urge.

While depression, anxiety, obsessive-compulsive symptoms, ADHD symptoms, and tics were each independently associated with overall HRQOL, their impact varied by HRQOL domain. Anxiety and depression were most robustly associated with psychological HRQOL, tic severity with physical HRQOL, and ADHD symptoms and depression with cognitive HRQOL. Given the lack of a valid linear model for the obsessive-compulsive domain of the GTS-QOL, no firm conclusions can be drawn from that particular regression analysis. It is notable that, in the correlational analysis, obsessive-compulsive symptoms were the clinical factor most closely corresponding with GTS-QOL Obsessive-Compulsive score. The association of specific psychiatric symptoms with specific HRQOL domains further validates the dimensional structure of the GTS-QOL scale.

Collectively, the battery of scales employed in this study explained 79% of the variance in GTS-QOL total score. Replication of these findings in another adult sample with chronic tic disorder is necessary to validate the regression model, but results nonetheless emphasize the importance of systematically evaluating for common psychiatric comorbidities given their individual and combined influence on HRQOL in this population. Consistent with this, recent practice guidelines prioritize recognition and management of psychiatric comorbidities, in addition to treatment of tics (60, 61). Given the multifaceted nature of the TS phenotype, disease-specific HRQOL measures, such as the GTS-QOL, are valuable means of quantifying patient-perceived overall burden from various aspects of the disorder. The GTS-QOL is specifically designed to measure the QOL impact of distinct motor and neuropsychiatric symptoms prevalent in tic disorder populations (16, 39, 62, 63). Generic QOL scales, on the other hand, tend to be less sensitive and/or comprehensive in their survey of the domains central to tic disorders (16, 62, 63). Strong consideration should be given to incorporating HRQOL scales, such as the GTS-QOL, as endpoints in clinical care and clinical trials. Longitudinal studies including such measures will be critical to elucidate risk factors for poor HRQOL in both children and adults with chronic tic disorders. The detrimental impact of psychiatric symptoms and tics on HRQOL in patients of all ages is evident (63), but additional work should also investigate broader psychosocial determinants of and potential protective factors for HRQOL, such as family dynamics, peer relationships, and school/work environment (13).

Our study has several notable limitations. First, participants were recruited from a tertiary care clinic and were predominantly white, limiting generalizability of study findings to other populations. Second, we relied on self-report measures of psychiatric symptoms rather than gold standard, clinician-administered rating scales. Third, the current sample size precluded more sophisticated modeling approaches, such as inclusion of interaction terms and medication status. Recruitment of a larger sample is in progress. Fourth, despite the scope of the assessment battery, we did not evaluate for symptoms specific to schizophrenia, autism spectrum disorder, or less frequent psychiatric conditions in TS. The fact that the constructed multivariable linear regression model explains the vast majority of GTS-QOL variance in this study suggests that no substantial variable omission occurred; however, unmeasured symptoms remain potential confounds that warrant consideration in future studies.

In conclusion, depression, anxiety, ADHD symptoms, and obsessive-compulsive symptoms are more strongly associated with HRQOL than tics in adults with chronic tic disorder. Systematic assessment of psychiatric comorbidities is imperative for optimizing clinical care and clinical research.

## Data Availability Statement

The dataset discussed in this article is not readily available as the authors do not have current IRB approval to share de-identified data from this study. Requests to access the dataset should be directed to david.a.isaacs@vumc.org.

## Ethics Statement

The studies involving human participants were reviewed and approved by Vanderbilt University Medical Center Human Research Protections Program. The patients/participants provided their written informed consent to participate in this study.

## Author Contributions

DI conceived and designed the study, implemented the protocol, and drafted the manuscript. HR and DC provided critical aid to study design and realization and provided substantive critique of the manuscript. All authors contributed to the article and approved the submitted version.

## Conflict of Interest

The authors declare that the research was conducted in the absence of any commercial or financial relationships that could be construed as a potential conflict of interest.
